# 

**Published:** 1920-07-24

**Authors:** 


### Matrons' and Sisters' Appointments.

Palmer Memorial Hospital, Jarrow-on-Tyne.—Miss Sarah G. Dalziel has been appointed matron. She was trained at Kilmarnock Infirmary, and has since been charge nurse at the Hull City Hospital, and matron of the Fusehill War Hospital, Carlisle.

Booth Hall Infirmary for Children, Blackley, Manchester.—Miss Winifred A. Arber has been appointed night superintendent. She was trained at Paddington Infirmary, and has since been ward sister at Cox Heath Infirmary and at Booth Hall Infirmary, Blackley, Manchester. Miss Arber holds the certificate of the Central Midwives Board.

Queen's Hospital, Birmingham.—Miss Adelaide Mary Jeffrey has been appointed night superintendent. She was trained at Western Infirmary, Glasgow, where she was later sister. She has since been ward and night sister at Bethnal Green Military Hospital. She holds the certificate of the Central Midwives Board.

Cameron Hospital, West Hartlepool.—Miss E. Corfield has been appointed sister. She was trained at the Guest Hospital, Dudley, and has since been staff nurse and holiday sister at Bradford Royal Infirmary. Miss E. Carries has also been appointed sister. She was trained at Gloucester Royal Infirmary, where she was later holiday sister.

### General Hospital, Birmingham.

### The College of Nursing (Limited by Guarantee).

### LONDON CENTRE.

For announcement concerning classes on Voice Training see "Round the Hospitals," page 434.

### BIRMINGHAM THREE COUNTIES CENTRE.

On Tuesday, July 27, in the Lecture Theatre of the General Hospital, Birmingham, at 5.30 p.m., Dr. J. B. Dixon, Tuberculosis Officer to the City of Birmingham, will lecture on "Tuberculosis." Entrance, members free; non-members fee, 1s.

### Hospital Sunday, 1920.

Up to July 16 £84,000 had been received at the Mansion House. This includes £764 from St. Columba's (Church of Scotland), Pont Street; £650 from St. Mark's, North Audley Street; £274 from St. James's, Paddington; and £200 from the Brixton Brotherhood.

